# Analysis of Multichannel EEG Patterns During Human Sleep: A Novel Approach

**DOI:** 10.3389/fnhum.2018.00121

**Published:** 2018-03-27

**Authors:** Patrick Krauss, Achim Schilling, Judith Bauer, Konstantin Tziridis, Claus Metzner, Holger Schulze, Maximilian Traxdorf

**Affiliations:** ^1^Department of Otorhinolaryngology, Head and Neck Surgery, Experimental Otolaryngology, Friedrich-Alexander University Erlangen-Nürnberg (FAU), Erlangen, Germany; ^2^Department of Otorhinolaryngology, Head and Neck Surgery, Friedrich-Alexander University Erlangen-Nürnberg (FAU), Erlangen, Germany; ^3^Department of Physics, Biophysics Group, Friedrich-Alexander University Erlangen-Nürnberg (FAU), Erlangen, Germany

**Keywords:** sleep stage analysis, EEG, spatially distributed cortical activity patterns, root-mean-square amplitudes

## Abstract

Classic visual sleep stage scoring is based on electroencephalogram (EEG) frequency band analysis of 30 s epochs and is commonly performed by highly trained medical sleep specialists using additional information from submental EMG and eye movements electrooculogram (EOG). In this study, we provide the proof-of-principle in 40 subjects that sleep stages can be consistently differentiated solely on the basis of spatial 3-channel EEG patterns based on root-mean-square (RMS) amplitudes. The polysomnographic 3-channel EEG data are pre-processed by RMS averaging over intervals of 30 s leading to spatial cortical activity patterns represented by 3-dimensional vectors. These patterns are visualized using multidimensional scaling (MDS), allowing a comparison of the spatial cortical activity patterns with the conventional visual sleep scoring system according to the American Academy of Sleep Medicine (AASM). Spatial cortical activity patterns based on RMS amplitudes naturally divide into different clusters that correspond to visually scored sleep stages. Furthermore, these clusters are reproducible between different subjects. Especially the cluster associated with the REM sleep stage seems to be very different from the one associated with the wake state. This study provides a proof-of-principle that it is possible to separate sleep stages solely by analyzing spatially distributed EEG RMS amplitudes reflecting cortical activity and without classical EEG feature extractions like power spectrum analysis.

## Introduction

Sleep stage classification is largely based on electroencephalogram (EEG) frequency band analysis, supplemented by detection of eye movements electrooculogram (EOG) and submental EMG. In 1937 sleep was first classified into five different stages (A to E) by Loomis et al. (1937) based on different EEG patterns. Recordings of brain waves were then concentrated on visibly recognizable patterns of cerebral activity in non-rapid eye movement (NREM) sleep. Originally, only the wave patterns of alpha and delta frequency band activity and isolated graphic elements such as K-complexes, spindles, vertex waves and posterior occipital sharp transients were identified. Rapid eye movement (REM) associated with respiratory and cardiac events was first described in 1953 and later formally combined to form the additional sleep stage REM (Aserinsky and Kleitman, 1953).

As the reliability of individual interpretations of EEG patterns was relatively low, a standardized scoring manual for the classification of human sleep was established in 1968 (Rechtschaffen and Kales, 1968). Its merits lay in the introduction of standardized terminology and technology, and a sleep classification system that was used worldwide for almost 40 years. A further scoring manual was not published until 2007, when the American Academy of Sleep Medicine (AASM) produced a more comprehensive manual taking into account the latest developments in sleep medicine, including additional measurement categories and sleep-related phenomena, as well as evolutionary sleep changes (Silber et al., 2007). The relevance of spatial EEG patterns in the interpretation of the vigilance level in extension of the classical frequency band analysis is reflected in the current AASM manual by the modification of the recommendation for electrode placement (F_4_-M_1_, C_4_-M_1_, O_2_-M_1_) and is the gold standard for sleep stage classification to date (Iber et al., 2007).

Nevertheless, there is a widely-held consensus that a classification of the different stages of the sleep-wake cycle based on the classic EEG frequency band analysis is still largely artificial (Uchida et al., 1992; Younes et al., 2015). Unfortunately, although rather successful on healthy subjects, existing approaches to automatically classify sleep stages based on EEG data so far do not work satisfactorily in patients (Boostani et al., 2017). Hence, it would be desirable to have alternative features to classify sleep stages. Although, there exist attempts to exploit spatial differences of sleep spindles (Cox et al., 2017), we here demonstrate that spatially distributed cortical activity, reflected by effective (i.e., root-mean-square, RMS) EEG amplitudes across the different recording channels, already includes all the relevant information for differentiating the sleep stages. We believe that this new, alternative approach of sleep stage classification, besides classical EEG feature extractions like power spectral density (PSD) analysis, will allow for the development of a fully automated and hence fully objective sleep stage analysis with even higher temporal resolution than the standard 30 s intervals in the future.

## Materials and Methods

### Database

The study was conducted in the Department of Otorhinolaryngology, Head & Neck Surgery, of the Friedrich-Alexander University Erlangen-Nürnberg (FAU) between March 2016 and November 2016, following approval by the local Ethics Committee (# 323_16 Bc). All 40 participating subjects, 27 male and 13 female, mean age 32.5 (±11.5) years, were recruited by the Department of Otorhinolaryngology, Head and Neck Surgery. Written informed consent was obtained from the participants before the cardiorespiratory polysomnography (PSG). Inclusion criterion for this study was age between 21 and 80 years. Exclusion criteria were a positive history of misuse of sedatives, alcohol or addictive drugs and untreated sleep disorders. Data analysis was carried out during “time in bed” of the subjects, accumulating to a total recording time of 310 h, 14 min, 30 s (mean recording time/time in bed per subject: 7 h, 34 min).

The mean fractions of total sleep time of the individual sleep stages (REM, N1, N2, N3; see below) were not different over the subjects’ age. This conclusion was drawn from the fact that, first, no linear correlations were found for the sleep stage fractions of total sleep time and the subjects’ age (always *p* > 0.05). Second, the subject cohort was separated at the median age (45 years) into a “younger” and “older” group. Again, no significant differences in sleep stage fractions of total sleep time could be found (Student’s *t*-tests, always *p* > 0.05, see Table 1). As revealed by 2-factorial ANOVA (age group × sleep stage; factor sleep stage: *F*_(3,116)_ = 80.45, *p* < 0.001), age groups did not differ from each other with respect to sleep stage fractions (*F*_(1,116)_ < 0.001; n.s.). In addition, Tukey *Post hoc* tests revealed (*F*_(3,116)_ = 1.51; *p* = 0.21) that N2 had the largest proportion (about 45%) of the total sleep time in both age groups (always *p* < 0.001).

**Table 1:** Relative fraction of sleep stages in percent.

Sleep stage	≤45 years (Median age) (± 95% CI)	>45 years (Median age) (± 95% CI)
REM	19.08 (±4.38)	17.45 (±4.22)
N1	14.33 (±2.71)	18.96 (±6.75)
N2	43.18 (±6.54)	46.32 (±5.83)
N3	23.40 (±7.96)	17.26 (±5.82)

### Cardiorespiratory Polysomnography (PSG)

Cardiorespiratory PSG was carried out using the 33-channel cardiorespiratory SOMNOscreen diagnostic system (SOMNOmedics, Randersacker, Germany). The technical implementation of the PSG followed the recommendations of the AASM using standardized procedures including an EEG, right and left EOG, electromyogram (EMG) of the mentalis and tibialis muscles, a nasal respiratory flow sensor (nasal pressure cannula), thoracic and abdominal respiratory effort sensors (induction plethysmography), body position sensors, pulse oximetry, snoring microphone, a one-channel ECG and an infrared video recording (Iber et al., 2007). Concerning recordings of EEG data, the following EEG derivations were used: F_4_-M_1_, C_4_-M_1_, O_2_-M_1_. Impedances were kept below 5 kΩ and data were sampled at 256 Hz with high-pass and low-pass filters at 0.2 Hz and 35 Hz, respectively. Minimal digital resolution was 16 bits per sample. ECG-signal elimination was performed for M1. The sleep stages (wake, N1, N2, N3, REM) were analyzed and scored visually in 30 s epochs as well as associated events according to the AASM criteria (Version 2.1, 2014) by a sleep specialist accredited by the German Sleep Society (DGSM; Iber et al., 2007; Berry et al., 2012). Thereby, typical artifacts (Tatum et al., 2011) have been marked and removed subsequently for further analysis and processing steps.

### Analyzing Spatiotemporal Patterns of Neuronal Activity: General Approach, Pre-Processing and Visualization of EEG Data

**Figure 1:**
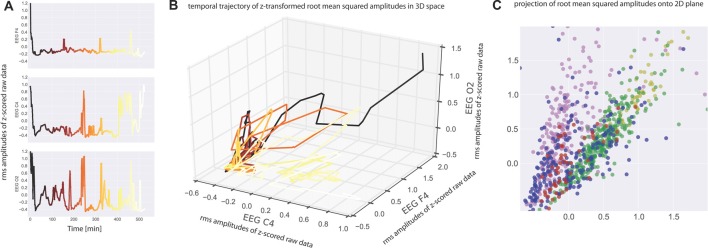
**(A)** Electroencephalogram (EEG) data recorded from one subject at three electrode positions (top panel: C_4_, middle panel: F_4_, bottom panel O_2_). Root mean square (RMS) values within 30 s bins of the z-scored raw recording data are plotted as a function of time. Note that the peaks correspond to wake states. The RMS values serve as coordinates for the three-dimensional plot of the data given in **(B)**. Colors from black to yellow denote the progression of time in both **(A,B)**. Note that each point in **(B)** represents a spatiotemporal activation pattern across the three recording electrodes, and the trajectory through these points reflects the development of these patterns over time. **(C)** For visualization purposes, data were projected from 3-dimensional space onto a 2-dimensional plane by means of multidimensional scaling (MDS) such that all mutual Euclidean distances in high-dimensional (3D) space, reflecting pattern dissimilarities, are preserved in the target (2D) space. Colors now depict sleep stages as classified via visual sleep scoring by an accredited medical sleep specialist: red: REM; blue: N1; green: N2; yellow: N3; pink: awake. Absolute coordinates of points have no particular meaning other than scaling relative distances between any pair of points, whereby distance reflects pattern dissimilarity.

Neurophysiological recording methods provide a measure of neuronal activity that evolves over time. We may therefore view such data as a time series of amplitude values, and in case of multichannel recordings like EEG this separately applies to each recording channel (Figure 1A). Consequently, the amplitude values recorded across the different recording channels during the same time bin reflect a spatial pattern of neuronal activity present at that time. In case of three recording channels as in our EEG measurements such patterns may be visualized as points in three-dimensional space, and the evolution of the recorded pattern over time forms a trajectory in three-dimensional space (Figure 1B). Note that distance of points within three-dimensional space is a measure of dissimilarity of patterns. For the analysis of spatial EEG patterns in each single subject the recorded potentials of the three EEG channels (F_4_-M_1_, C_4_-M_1_, O_2_-M_1_) were considered as the dimensions of a 3-dimensional vector: raw data (amplitude values) within each EEG recording channel (C_4_-M_1_ (for x-axis), F_4_-M_1_ (for y-axis), O_2_-M_1_ (for z-axis)) were z-scored (i.e., normalized to zero mean and unit variance) and subsequently binned in 30 s intervals by RMS averaging (Figure 1A; Colors from black to yellow denote the progression of time). The RMS amplitudes of the three recording channels correspond to a 3-dimensional vector for each 30 s interval, whereas successive vectors form a trajectory in 3-dimensional space (Figure 1B; Colors as in Figure 1A).

For easier visualization we may then project those points from three-dimensional space onto a two-dimensional plane by means of multidimensional scaling (MDS). MDS is used to project points from a source space onto a lower dimensional target space such that all mutual Euclidean distances, reflecting dissimilarities of EEG patterns, are preserved (Torgerson, 1958; Kruskal, 1964a,b; Figure 1C).

Subsequently each point (epoch) in the 2D-plot is assigned to the individual epoch of sleep stage (W, N1, N2, N3, REM) which was classified by visual sleep stage scoring. Visual sleep stage analysis was blinded to the results of MDS. In addition, for a clear graphical presentation each point (representing a single epoch) was color coded (REM sleep (red), N1 (blue), N2 (green), N3 (yellow), W (pink)). Points (epochs) of the same color correspond to the same sleep stage. Ideally five color-separated point clouds will be the result. As can be seen in Figure 1C, the spatiotemporal activity patterns recorded during the different sleep stages form clusters of points rather than “color noise”, that is, neuronal activity patterns recorded during sleep are more similar within than between sleep stages. Based on these similarities and dissimilarities we may now compare the clusters of points associated with the different sleep stages.

### Multidimensional Cluster Statistics

We applied a recently developed method to statistically compare clusters of data points in n-dimensional space. The method allows determining whether overlapping clusters are significantly different from each other. It is based on calculating the so called discrimination value taking into account all intra- and inter-cluster distances between each pair of data points in n-dimensional space. Those distances are a measure of how similar or dissimilar two points in n-dimensional space are. The more similar they are, the smaller their distance is.

Subsequently a number of permutations of the points’ labels are performed (i.e., random re-labeling) and intra- and inter-cluster distances are re-computed resulting in a new discrimination value for each permutation. The distribution of these discrimination values is used to estimate a *p*-value that indicates if two given clusters are separated significantly. The method is described in detail in Krauss et al. (2016).

## Results

### Separation of Sleep Stages Based on Spatially Distributed EEG Patterns

**Figure 2:**
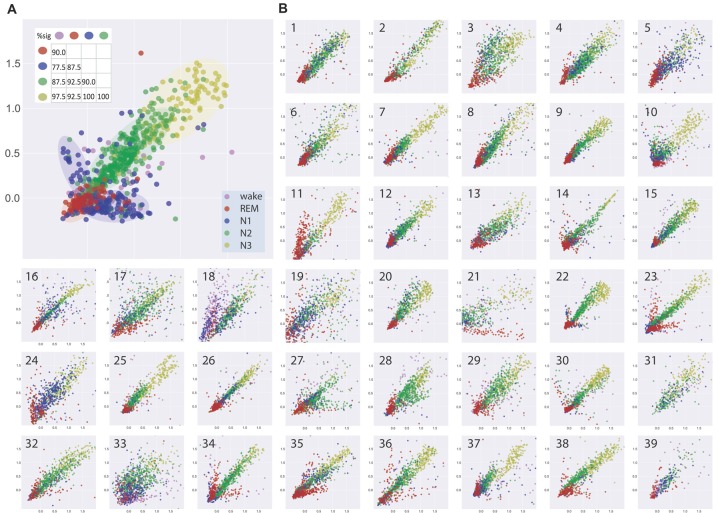
**(A)** Visualization of similarities and dissimilarities of sleep stage associated EEG patterns of one exemplary subject. EEG patterns associated with particular sleep stages form clusters of points which shift along a systematic, ordered gradient, from REM sleep to N1 to N2 to N3. Results of multidimensional cluster statistics (MCS) are summarized in the inset. The numbers in the matrix correspond to the percentage of significant *p*-values of the pairwise comparisons of individual subjects’ sleep stage clusters. Note, that a significant *p*-value in MCS indicates that two given clusters are significantly different from each other despite a possible overlap. Absolute coordinates of points have no particular meaning other than scaling relative distances between any pair of points, whereby distance reflects pattern dissimilarity. **(B)** Same type of analysis for 39 further subjects.

Figure 2A shows the visualization of EEG patterns during sleep for one exemplary subject. Obviously, the EEG patterns during different sleep stages formed clusters of points in clearly separable regions (see color shaded areas), although there still was some overlap. Interestingly, these clusters of similar neuronal activity patterns associated with particular sleep stages were not randomly distributed across the two-dimensional plane but rather form a systematic gradient, from REM sleep to N1 to N2 to N3.

Figure 2B shows the same type of analysis for 39 further subjects. Although there may be considerable variability between subjects, the general systematic described for the subject in Figure 2A was again present in almost all subjects. Exceptions could be a higher degree of scattering of the points within a cluster, reflecting higher variability of EEG patterns during a sleep stage and or higher degree of overlap between EEG patterns associated with different sleep stages (see e.g., Figure 2B, panels 19, 33). Other subjects showed highly homogeneous and separable clusters of EEG patterns (see e.g., Figure 2B, panels 14, 22, 26). In one subject where REM sleep was missing (Figure 2B, panel 31), the systematic relation of EEG patterns between the NREM sleep stages was nevertheless preserved.

We analyzed the data with multidimensional cluster statistics (MCS; “Materials and Methods” section, Krauss et al., 2016). The results are summarized in Figure 2A inset. The numbers in the matrix correspond to the percentage of *p*-values of the pairwise comparison between different sleep stage clusters for the individual subjects that became significant (*p* < 0.05). A significant *p*-value in MCS indicates that two given clusters are significantly different from each other, despite possible overlap.

**Figure 3:**
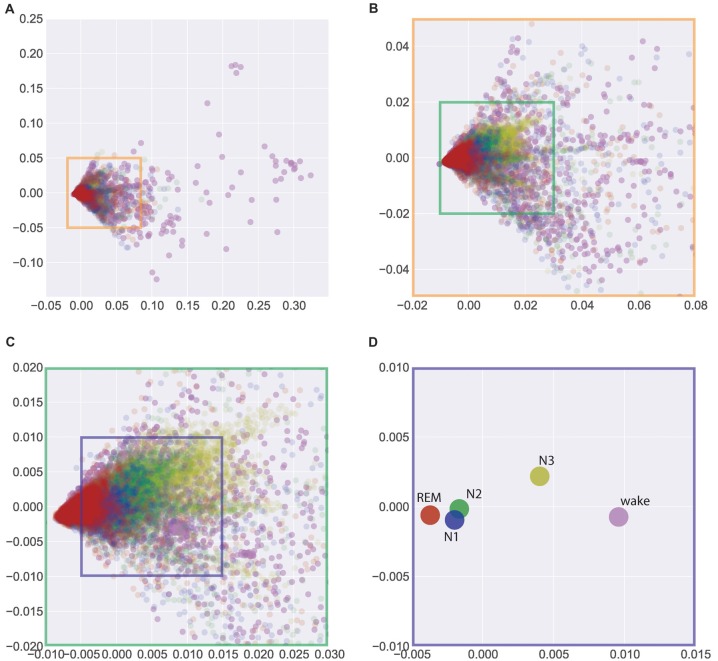
Visualization of similarities and dissimilarities of sleep stage associated EEG patterns across all 40 individual subjects. **(A)** Total overview encompassing all 37229 data points (wake and sleep stages). **(B,C)** Zoom into the data shown in **(A)** to emphasize the sleep stages only. **(D)** Centroids of clusters given in **(A)**. The systematic shift of clusters and centroids demonstrates the interindividual similarity of the sleep stage associated EEG patterns. Absolute coordinates of points have no particular meaning other than scaling relative distances between any pair of points, whereby distance reflects pattern dissimilarity.

In search for a general, interindividual systematic of EEG patterns, we pooled the data across all 40 subjects (from Figures 1C, 2) in Figure 3. Figure 3A gives a complete overview across all 37229 data points (i.e., periods of 30 s of EEG recording) obtained from the 40 subjects, thereby allowing for a comparison of sleep associated EEG patterns with those measured during the wake state. Obviously, EEG patterns during wakefulness are much more heterogeneous than those during all sleep stages. Figures 3B,C show zooms into the plot given in Figure 3A, so that the display window in Figure 3C roughly corresponds to those given in Figure 2, focusing on the sleep stages only. The described systematic relation between sleep stage associated EEG patterns that could already be seen for individual subjects—despite considerable interindividual variation—was again obvious in the pooled data across subjects: Again, a systematic shift of clusters can be seen from REM sleep (red) to N1 (blue) to N2 (green) to N3 (yellow). This is even more obvious when the centroids of the different clusters of the pooled data—so to say the “prototypical” EEG patterns for a given sleep stage—are plotted, as shown in Figure 3D. Obviously this systematic relation between EEG patterns associated with the different sleep stages reflects an overall principal that is invariant across the subjects’ individual brains.

Unexpectedly, we found the largest difference between the EEG patterns associated with REM sleep and those recorded during the wake state, although the respective EEG traces during wake and REM sleep periods look very similar (both show low-voltage, desynchronized brain waves in the alpha range and above, see “Discussion” section). On the other hand, the smallest differences were seen between “prototypical” EEG patterns of N1 and N2.

**Figure 4:**
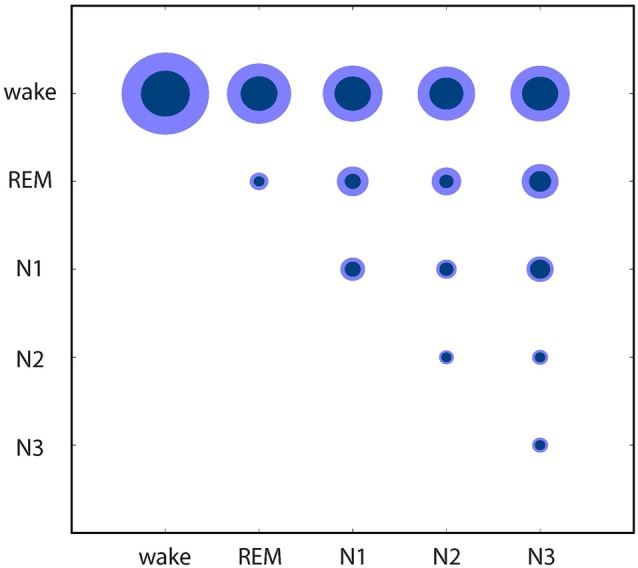
Quantitative analysis of similarities and dissimilarities between the EEG patterns associated with different sleep/wake stages. Circle diameters represent mean distances between points (inner circles) and standard deviation (outer circles), both within clusters (on the diagonal of the plot) and between clusters (off the diagonal) of the individual recordings presented in Figures 1C, 2.

Finally, these relations between the EEG patterns associated with different sleep/wake stages are further quantified in Figure 4: based on the individual data from Figures 1C, 2, 4 shows mean distances between points (inner circles) and standard deviation (outer circles), both within clusters (intra-cluster distances; on the diagonal of the plot) and between clusters (inter-cluster distances; off the diagonal). This quantitative analysis confirms that indeed the wake state displays the highest variability of EEG patters (large intra-cluster point distances), and that these patterns also show the biggest differences to all sleep stages (top row in Figure 4). Furthermore, REM sleep is characterized by very homogeneous EEG patterns (i.e., small intra-cluster point distance), as are patterns from N2 and N3. Nevertheless, the inter-cluster differences are larger between REM and NREM sleep stages (N1, N2, N3), demonstrating the EEG patterns during the different NREM sleep stages are more similar to each other than to those during REM sleep.

## Discussion

When visual sleep stage analysis was already being optimized in 2007, awareness of the spatial organization of specific EEG graphic elements (K complexes and delta activity in the frontal leads, sleep spindles in the central leads, and alpha rhythm over the occipital cortex) led the AASM to change the earlier recommendation on electrode configuration made by Rechtschaffen and Kales (McCormick et al., 1997; Werth et al., 1997; De Gennaro et al., 2000; Happe et al., 2002; Iber et al., 2007). Building on this, the present study investigated spatially distributed EEG patterns based on RMS amplitudes and correlated them with the current gold standard (visual sleep stage analysis according to AASM). We were able to provide the proof-of-principle that sleep stages can be differentiated only on the basis of RMS amplitudes across three EEG recording channels (F_4_-M_1_, C_4_-M_1_, O_2_-M_1_) and without using classic EEG frequency band analysis, template matching or wavelet analysis.

Beyond that, this novel approach even allows for both qualitative and quantitative evaluations of sleep architecture: Qualitatively, the homogeneity or heterogeneity of spatiotemporal EEG patterns of a given sleep stage, and the similarity or dissimilarity of such patterns between different sleep stages can objectively be evaluated.

In the present report, this comparison yielded two important insights into the relation between EEG patterns associated with different sleep/wake stages: First, we found the least dissimilarity between the EEG patterns associated with N1 and N2. By definition, a main difference between N1 and N2 is the existence or absence of K-complexes and sleep spindles (Rechtschaffen and Kales, 1968; Iber et al., 2007), whereas the ongoing EEG activity (low-amplitude mixed frequency) in both cases is the same. Therefore it seems plausible that the EEG patterns we found during N1 and N2 do not differ much. This fact is even more understandable by looking at the outdated 3 min rule of the Rechtschaffen and Kales manual, where the occurrence of a spindle or K-complex defines the following 6 epochs (3 min) as N2 rather than N1, even if no further spindle of K-complex was detected (Rechtschaffen and Kales, 1968). One therefore may discuss if N1 and N2 really are separate sleep stages or rather subtypes of the same sleep stage. On the other hand, as our method may equally be applied to EEG data with more than three recording channels, it may open the opportunity to provide a more detailed description of sleep stages which could possibly reveal further subtypes of sleep stages. Nevertheless, the fact that it is already possible to discriminate between the classical sleep stages based on EEG patterns of RMS amplitudes only strongly emphasizes the analytical power of our new approach.

A somewhat surprising result of our study was the large difference between EEG patterns associated with wake vs. REM sleep stages. Based on animal studies, EEG recordings between these two states are considered to be virtually indistinguishable by eye (see e.g., Corsi-Cabrera et al., 2001; Horne, 2013), and also for human sleep the two stages have more in common than features that separate them (for detailed review see Matarazzo et al., 2011; Siegel, 2011; Horne, 2013). Therefore our tool seems to provide a new, easy and reliable method to separate REM sleep from wake.

Regarding the limitations of the study, we think that despite a clear terminology as well as detailed technical specifications and scoring rules, visual sleep stage analysis still has the problem of substantial interrater reliability. Numerous studies have already dealt with this topic and report inter-expert agreements (Cohens kappa; κ) in sleep stage classification of 0.65–0.78 (Danker-Hopfe et al., 2004, 2009; Pittman et al., 2004; Anderer et al., 2005; Malinowska et al., 2009; Magalang et al., 2013; Wendt et al., 2015). Especially NREM sleep stages N1 and N2 seem to show only a fair to moderate observer agreement with a *κ* = 0.31–0.46 and *κ* = 0.60 respectively (Danker-Hopfe et al., 2004, 2009; Magalang et al., 2013). Although the observers in this study have many years of experience in the visual scoring of sleep EEG data and have been accredited by the DGSM, this limitation certainly also applies to our study. However, since the visual evaluation continues to be the gold standard, this limitation will not be avoidable for the time being. On the other hand, this may explain the least dissimilarity between the prototypical EEG patterns of N1 and N2 as seen in Figure 3.

Another limitation of this study is that our proof-of-concept still has to be tested in patients with untreated sleep disorders, e.g., sleep disordered breathing.

We believe that in the future based on this new approach the common but artificial segmentation of EEG recordings into epochs of 30 s time intervals assigned to one single sleep stage could be overcome by a fully automated and hence fully objective method that could be used to perform sleep stage classification with much higher temporal resolution (i.e., assigning sleep stages to shorter time intervals than the common 30 s intervals). A resulting adaptive scoring system defining the start and end of each sleep stage without relying on discrete epochs has already been considered by the AASM Visual Scoring Task Force (Iber et al., 2007). One possible advantage of such an approach might be a more natural representation of sleep continuity including a more detailed impression of the microstructure of sleep fragmentation (Silber et al., 2007). In conclusion, we believe that this new approach of multichannel EEG pattern analysis based on RMS amplitudes will be a powerful supplement for the improvement of subsequent sleep stage classification based on e.g., machine learning and deep learning methods.

## Author Contributions

PK, MT and HS wrote the manuscript. PK and CM developed analysis methods. PK and AS prepared the figures and analyzed the data. JB and MT performed the experiments. PK, AS, KT, CM, HS and MT discussed the results.

## Conflict of Interest Statement

The authors declare that the research was conducted in the absence of any commercial or financial relationships that could be construed as a potential conflict of interest.
